# Proteogenomic Analysis of Bacteria and Archaea: A 46 Organism Case Study

**DOI:** 10.1371/journal.pone.0027587

**Published:** 2011-11-17

**Authors:** Eli Venter, Richard D. Smith, Samuel H. Payne

**Affiliations:** 1 Department of Informatics, J. Craig Venter Institute, Rockville, Maryland, United States of America; 2 Biological Sciences Division, Pacific Northwest National Laboratory, Richland, Washington, United States of America; Inserm U869, France

## Abstract

Experimental evidence is increasingly being used to reassess the quality and accuracy of genome annotation. Proteomics data used for this purpose, called proteogenomics, can alleviate many of the problematic areas of genome annotation, e.g. short protein validation and start site assignment. We performed a proteogenomic analysis of 46 genomes spanning eight bacterial and archaeal phyla across the tree of life. These diverse datasets facilitated the development of a robust approach for proteogenomics that is functional across genomes varying in %GC, gene content, proteomic sampling depth, phylogeny, and genome size. In addition to finding evidence for 682 novel proteins, 1336 new start sites, and numerous dubious genes, we discovered sites of post-translational maturation in the form of proteolytic cleavage of 1175 signal peptides. The number of novel proteins per genome is highly variable (median 7, mean 15, stdev 20). Moreover, comparison of novel genes with the current genes did not reveal any consistent abnormalities. Thus, we conclude that proteogenomics fulfills a yet to be understood deficiency in gene prediction. With the adoption of new sequencing technologies which have higher error rates than Sanger-based methods and the advances in proteomics, proteogenomics may become even more important in the future.

## Introduction

With the advance in sensitivity and computational power of high throughput proteomics, many datasets now offer expansive proteome coverage. A growing trend is to use these data to re-evaluate protein sequence predictions, i.e. proteogenomics [1], [2]. As most genome annotation pipelines consist of automated gene finding, they lack experimental validation of primary structure [3], [4]. Thus proteogenomics offers valuable opportunity to correct, corroborate, and supplement genomic predictions as an orthogonal data-type from DNA-centric evidences (e.g., sequence homology, transcriptome mapping, codon frequency, etc.). In a variety of organisms, new insight from proteogenomics has consistently improved genome annotation [5], [6], [7], [8], [9].

Fundamentally, an accurate primary structure implies knowing the correct start/stop coordinates of the protein, which may be erroneously predicted for 20% of genes in some bacterial and archaeal genomes [10], [11], as well as recognizing any true frame-shifting events, that should be distinguished from sequencing errors. A more advanced gene model should contain information about the mature protein sequence. For example, protein cleavage events such as N-terminal signal peptides are particularly valuable clues for protein localization in the prokaryotic cell. Similarly, characterizing a mature antimicrobial from the nascent pre-protein can add valuable information as to how such a protein assumes its biological role [12]. Furthermore, modifications to amino acids (e.g., phosphorylation) can implicate a protein in distinct and often transient biological processes (e.g., regulation of gene expression). None of these protein maturation events are observable via DNA sequencing.

Proteogenomics as a field has tended to utilize datasets generated from a single organism or biological system. Although various techniques have been explored to gain more proteome coverage [13] or to recover specific subsets [14] or target protein N-termini [15], little work to re-evaluate protein annotation has been performed with datasets from multiple organisms. Gupta et al. first explored the concept of comparative proteogenomics with the analysis of three different *Shewanella* [16]. A major finding of this work was that the confidence of ‘one-hit-wonder’ proteins could be increased by the observation of orthologs in a different species' proteomics dataset.

We have previously published a proteogenomics methodology for discovering novel protein coding regions using only a single organism, *Yersinia pestis* [17]. To ensure the generality of our approach, we tested it on 46 organisms from eight bacterial and archaeal phyla. This expansive diversity uncovered shortcomings and flaws observable only in certain datasets, and produced a more robust pipeline. Selected results are highlighted to describe the types of events that can be discovered, including new genes, conflicts with current genes, disputation of pseudogenes, and mature protein events. In total we reveal over 2000 new and corrected genes. We also looked for reasons why novel genes identified in this study may have been missed in the original genome annotations.

## Results

Proteogenomic analyses are defined by a search for novel protein coding regions in an organism's genome. To accomplish this, proteomic data is searched to identify sequences which are not currently part of the genome annotation. Tandem mass spectra are typically interpreted using a protein sequence database to facilitate peptide identification [18]. In our proteogenomic pipeline [17], peptide/spectrum matches (PSMs) are identified from a six frame translation of the genome, instead of simply the predicted protein sequences. Only highly confident identifications are kept; the default cutoff for our pipeline is an MSGF score of 1e-10 [19]. Peptide sequences are mapped to their genomic locus and grouped with other peptides within an open reading frame (ORF). Many proteogenomics methodologies do not perform any additional filtering and simply assert novel coding regions from all novel peptides. After testing our pipeline on numerous datasets, we have found that this is insufficient. We adapted our existing proteogenomics pipeline [17] to be functional across genomes varying in %GC, gene content, phylogeny, and genome size. Additionally, our pipeline has been tested on datasets with over 15 million tandem mass spectra and those with only ∼100,000, showing robustness regardless of proteomic sampling depth. Using 46 organisms (Table S1) with a wide variety of characteristics, we refined the data processing path, most notably FDR stringency and ORF filtering (see Methods for a full description).

### Pipeline Updates

As a first step towards improving the proteogenomics pipeline, we replaced the local false-discovery rate (lFDR) with the exact p-value of a PSM. An lFDR is a heuristic approximation of a pvalue, which bins PSMs by score and calculates the false-discovery of each bin [20]. This method is not robust for small or medium datasets where too few results of a given score range force the bin size larger, removing most of the intended discriminating power. Moreover, the pvalue as calculated by the scoring algorithm MSGF is PSM specific, and not a grouped approximation [19]. Finally, we have dramatically decreased the permissible FDR; the current default cutoff for MSGF is 1e-10 which typically results in a spectrum level FDR of 0.01% (peptide FDR∼0.3%).

Even at high PSM specificity (spectrum FDR<<0.01%), some of the proteogenomic predictions such as novel genes could be wrong. The need for filters which operate on open reading frames (not simply PSMs) was identified from the observation of multiple suspect coding regions at zero false-discovery. False-discovery of 0.0% was achieved by increasing MSGF stringency to 1e-13 or 1e-14 instead of 1e-10. In these tests zero spectra were identified with peptides from the decoy database. Therefore, all novel peptides were inspected as potentially confirming novel genes, not requiring two peptides per ORF. In multiple organisms, we observed proposed novel coding regions that completely overlapped currently annotated proteins with substantial peptide support (Figure S1). Additionally many of these ORFs lacked a start codon. Such ORFs were always represented by one peptide. As a result of these suspect coding regions which are present even at a presumed zero false-discovery, we found it necessary to utilize ORF filters (see Methods). ORF filters are not meant to overcome loose PSM filters, but rather to help identify putative coding regions that are likely false-positive even when all PSMs are highly confident. We previously used four ORF filters [17], however in the analysis of genomes with high GC, it was necessary to add a peptide length clustering filter. High GC genomes have many long open reading frames that are not genic. These long ORFs have a potential to contain multiple false-positive peptides, which would pass the two peptide filter. After modeling the interpeptide distance from all known proteins with proteomic data, we set a maximum interpeptide distance at 750 nucleotides. Using this length filter, we were able to remove many false-positive protein predictions in high GC genomes.

### Conflict Resolution

**Figure 1:**
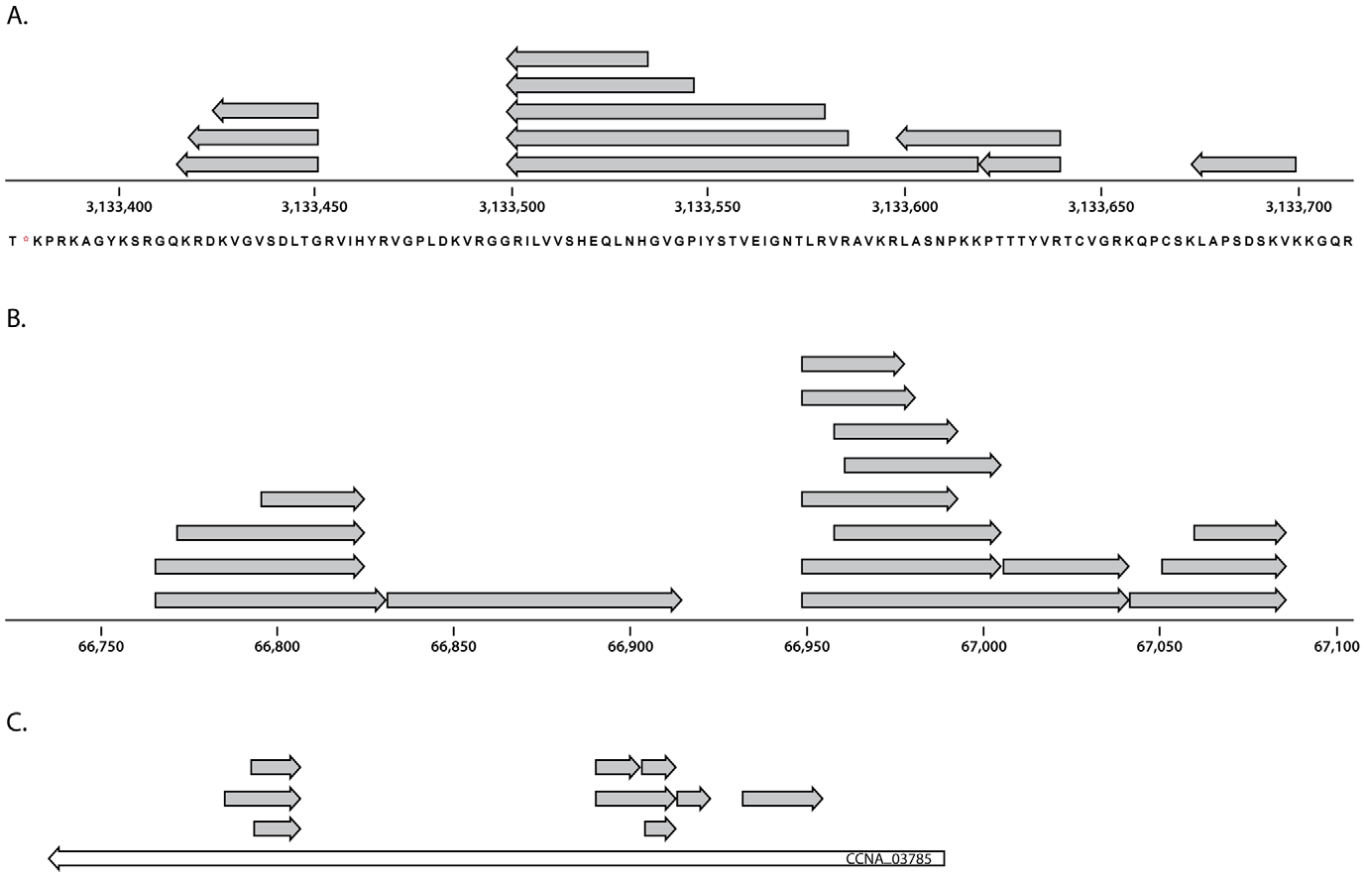
Novel coding content. Regions of a genome are shown where novel peptides (grey arrows) are not contained within a currently annotated protein. A, an unannotated region of *Geobacter sulfurreducens* corresponding to ribosomal protein S12. B, an unannotated region of *Rhodobacter sphaeroides* plasmid for which there are no blast matches. C, a level 3 conflict in *Caulobacter crescentus*. Currently annotated protein CCNA_003785 (white arrow) is dubious and should be replaced with a gene model on the opposite strand.

We further discriminate potential novel coding regions by characterizing their location relative to other genes. Of the over 2000 novel coding events we discovered, most are located in empty regions of the genome lacking any annotation. Regardless of whether the genes have strong homology to known proteins (Figure 1A and B), they can still be confidently added to a genome's annotation. However, complications can arise when proposed novel coding regions overlap current genomic features. As bacterial genomes have a very high coding density, new coding regions occasionally overlap with current annotations (e.g. utilizing a different frame of translation). Therefore, we created a “conflict report” to judge the accuracy and specificity of proposed proteogenomic corrections. Overlapping proteins were split into six categories, each with an *a priori* interpretation of biological feasibility (see Methods). Levels 1 and 2 consisted of protein overlaps under 40 bp, a common event in bacterial genomes [21], [22], and were not considered *conflicts*.

Conflict levels 3–6, which consisted of overlaps in excess of 40 bp, required more careful inspection. Conflict levels 3 and 4 had proteomic support (i.e. peptides) for only one of the genes involved in the overlap, and are differentiated by the *unsupported* protein. In a level 3 conflict, the non-observed protein is hypothetical (Figure 1C), and almost all level 3 conflicts arise from dubious annotation. Commonly, the genomic DNA at these loci contains multiple competing open reading frames, and the gene prediction algorithm failed to choose the correct frame. Although the number and type of conflicts were widely variable between datasets, level 3 conflicts were generally more common than levels 4, 5, or 6 (Table S2). A level 4 conflict occurred when the unobserved protein was named anything but ‘hypothetical’. Before stringent PSM and ORF filters, we encountered many level 4 conflicts. Based on our observations, a large number of level 4 conflicts were generally indicative of high protein FDR. Conflict levels 5 and 6 occurred when both overlapping proteins had proteomic support (see Methods). Again, most often these conflicts were signs of high protein FDR. Although two proteins rarely overlap by more than 40 nucleotides in bacteria [23], we have observed a few in high GC organisms.

The decline in conflict levels 4,5,6 as PSM stringency increases closely mirrors the decline in novel genes predicted. For example, in *Caulobacter* changing the MSGF cutoff from 5e-08 to 1e-10 changes the PSM FDR from 0.5% to 0.02% (peptide FDR 5% and 0.3% respectively). Coordinated with this increased stringency is a decrease in novel genes (195 to 25) and conflicts levels 4, 5, 6 (261 to 49). Moreover, at the stringent level, almost all remaining conflicts (45/49) are between current genes, not involving proposed novel genes. As a comparison, the number of protein identifications for current annotations decreases only marginally over this range, 2725 identifications to 2551. The decrease in conflicts (levels 4, 5, 6) coordinated with PSM stringency is a general principle (Table S3). On average when going from MSGF cutoff of 5e-08 to our default f 1e-10, the number of conflicts decreases 4 fold (mean 4.0, median 2.2). The number of novel proteins is also over inflated with less stringent FDR (mean 3.4× inflation, median 1.8×). This result shows two important things. First loose FDR leads to numerous false-positive predictions. Second, conflict classification as presented here shows good specificity in highlighting likely false-positives.

### Genome Annotation Deficiencies

In an effort to discover why novel genes identified in this study were missed during the original genome annotation we first looked at whether the omission is strictly a function of older annotation, i.e. have gene prediction algorithms solved the problem already? There was no correlation between the number of annotation corrections and date of the genome annotation or genome GC levels: correlation coefficient of −0.38 and 0.15 respectively (Figure S2, S3). Looking gene by gene at characteristics like %GC and codon usage, we did not find novel genes distinct or outside of the norm (Figure S4, S5). Although many of the novel genes were short, they were not below the cutoff for annotation (Figure S6).

We also looked at genome context for novel genes: those that overlapped an annotated gene, those that overlapped a pseudogene, and those that did not overlap any genomic feature. The relative proportion of these three groups varied widely by genome. For example, in *Caulobacter* almost all novel genes overlapped an annotated gene (level 3 conflict); however, in *Synechocystis*, all genes were simple novel genes, and did not overlap any genomic feature.

**Figure 2:**
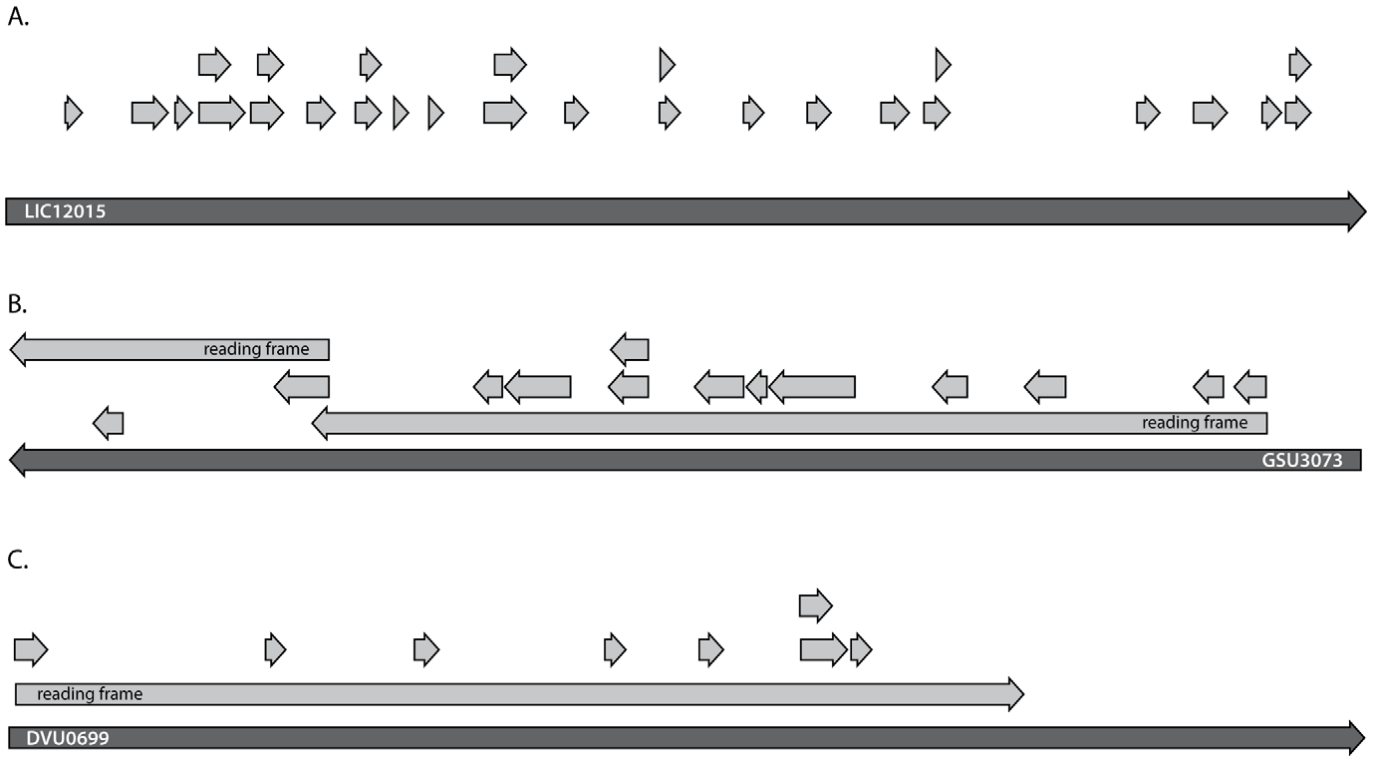
Classes of translated pseudogenes. Peptides (light grey arrows) are shown to map within pseudogene boundaries (dary grey arrows), proving that the region is translated to protein. A, peptides in a single coherent open reading frame spanning the entire length of the pseudogene LIC 12015 from *Leptospira interrogans*. It is unclear why such regions were designated as pseudogenes. B, peptides fall within two different translation frames which are located within *Geobacter sulfurreducens* pseudogene GSU3073. This situation can arise when the underlying genome sequence is erroneous and contains artificial indels, or if the two regions are separately translated. C, peptides fall in a single open reading frame which only partially covers the genomic regions annotated as a pseudogene, *Desulfovibrio vulgaris* pseudogene DVU0699. This confirms the conceptual translation of the pseudogene (i.e. ending in an in-frame non-sense codon).

In many of the genomes, peptides overlap a genomic region demarcated as a pseudogene. We observed at least three categories of pseudogene misannotation (Figure 2). In the first type of misannotation, peptides were identified in a single open reading frame spanning the entire start-stop of the predicted pseudogene (Figure 2A). As the peptides within this span make a coherent gene model without frameshifts or interruptions, it was unclear why these were annotated as a pseudogene. Figure 2B shows the murF locus of *Geobacter sulfurreducens*; peptides lie in two different frames of translation. Thus according to the straightforward interpretation of the genome, this cannot make a single protein. One possible explanation is that there is a DNA sequence error (indel) causing the apparent frame shift; a separate possibility is that there are two protein products at this locus, although there are no other examples of this gene being split. The third pseudogene misannotation shows the true conceptual translation of a pseudogene, where the reading frame ends before the gene model, Figure 2C. Thus the translation could be described as a partial gene product. The original Genbank annotation notes that the open reading frame is a partial hit to Pfam PF02518, a histidine kinase-like ATPase, but other protein family profiles support this sequence as containing the full domain (e.g. cd00075 from NCBI's CDD).

### In vivo cleavage - Signal peptides and lipoproteins

Many subcellular localization mechanisms utilize conserved sequence motifs that serve as molecular addresses and often involve enzymatic cleavage in proximity of the motif. This cleavage creates a new protein N-terminus that is amenable to discovery via proteomics.

**Figure 3:**
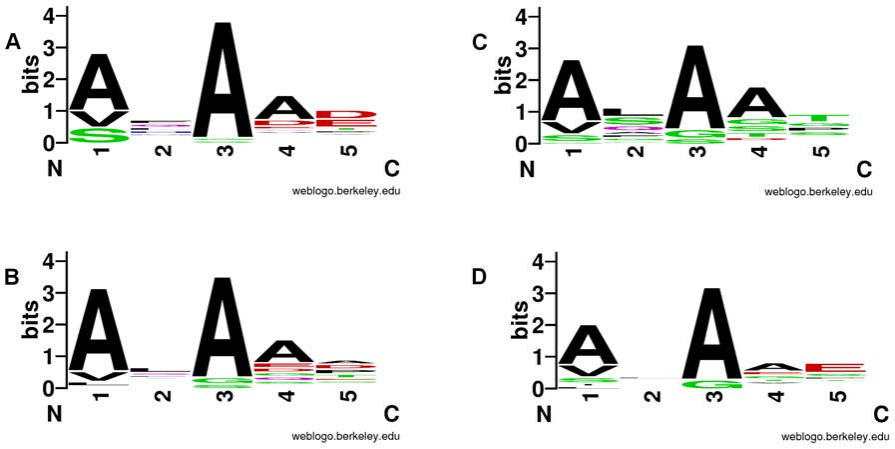
Signal peptidase I motifs. The amino acid residues surrounding signal peptidase cleavage sites are shown for four organisms. The five amino acid residues are three residues prior to cleavage (−3, −2, −1) and two residues post cleavage (+1, +2). A, *E. coli*; B, *Caulobacter crescentus*; C, *Deinococcus radiodurans*; D, *Cyanobacterium synechocystis*. Figures were created using weblogo.berkeley.edu with default parameters.

Proteins exported from the cytoplasm through the Sec-dependant pathway contain a ∼20 residue N-terminal sequence to target the protein to the membrane and mediate cleavage. The signal peptide contains three conserved motifs: early basic residue(s), a hydrophobic patch, and a three residue recognition motif for signal peptidase I [24]. We identify sites of signal peptide cleavage in proteomics data by finding the first peptide of a protein as non-tryptic on its N-terminus, and then requiring the three conserved motifs. Requiring these three elements filters the proposed signal peptide set by 20–80% as compared to previous methods [9], greatly increasing specificity. We identified 1175 sites of signal peptide cleavage. By aligning the three residues prior to cleavage (−3, −2, −1) with the two following cleavage (+1, +2), we determined the observed signal peptidase I recognition motif by taxa (Figure 3). In general, the motif is similar between taxa and alanine is expectedly prominent at residues −1 and −3. Previous reports of bacterial signal peptides have uniformly reported the ‘AxA’ motif for signal peptidase I cleavage [25], [26].

We observed additional maturation signals in proteins that contained a non-tryptic first peptide, but failed to contain at least one of the three signal peptide criteria. In *Arthrobacter*, many proteins lacked the signal peptidase I recognition motif, but instead contained the common sequence L-x-G/A-C, the lipoprotein signature. A final type of N-terminal maturation was N-terminal methionine excision, or NME. An exceptionally large fraction of proteins lacking a hydrophobic patch had methionine immediately prior to the first peptide (e.g., 45% for *Caulobacter* and 55% for *Cyanothece*). In most of these instances the first peptide started with alanine or other residues consistent with NME [27], suggesting that the protein is predicted too long.

## Discussion

Although intense effort has gone into determining the correct functional annotation of proteins [28], primary gene structures are still imperfect. Proteomics provides a powerful experimental data type to access and improve the quality of genome annotation. A key advantage is the direct correlation between protein annotation and a protein based assay. In this study, analysis of 46 genomes spanning eight bacterial and archaeal phyla across the tree of life allowed us to develop a robust approach for proteogenomics annotation that is functional across genomes varying in %GC, gene content, proteomic sampling depth, phylogeny, and genome size. In proteogenomics, specificity proves more important than sensitivity and leniency at the hopes of greater genome coverage can dramatically increase the chance for false-positive novel protein identification. We evaluate the quality of proposed proteogenomic corrections through the conflict report. By no means implying that overlapping proteins are not real or cannot be found by proteogenomics [29], the vast majority of novel proteins with significant overlap were typically low quality and weeded out by stringent filters.

Our effort to understand why genes are missed in the initial annotation revealed that the only consistent problem was the expected sensitivity/specificity decline for short proteins. Citing the diversity of other errors, we suggest that all genome annotations leverage proteomics, either through concurrent proteomics/genomics sampling, or by utilizing the compendium of proteomically verified ORFs as a part of their extrinsic evidence set (i.e. in addition to blast or hmm searches).

For pseudogenes, we showed three types of misannotation, each resulting from a different deficiency in the sequencing and annotation process. Resolving the annotation of these is difficult, partially attributable to the potential for genome sequence errors. More pointedly, there is not a consensus on the meaning of ‘pseudogene’, whether ‘non-functional’ applies to the translated product's biochemical function or to the ability of a genomic locus to produce a viable transcript which gets translated. While this discussion is outside of the scope of this work, our perspective as proteomic scientists is that all translated products should be included in common database downloads.

We focused largely on false-negative annotations, where a region of DNA was not assigned to be protein-coding, but should have been. A more difficult misannotation is false-positives, which we find as novel/dubious pairs in the data and are more apparent for some genomes. These dubious genes can have far reaching effects, as they propagate through future genome annotations in what is known as “transitive disaster”.

As a final part of our methodology, we analyze the datasets to discover in vivo protein cleavage. Proteomic determination of cleavage sites offers several distinct advantages over strictly computational approaches that predict cleavage events directly from sequence. In addition to providing experimental validation of cleavage, proteomics yields a broad and unbiased sample of cleaved proteins. For example, in the *Geobacter* data sets over 150 proteins were identified as having a signal peptide, yet the overlap between these three genus members was only nine proteins. Thus a large number of distinct proteins were identified. This diverse set could serve as a powerful training/testing set to improve computational tools.

## Materials and Methods

The pieces of the data processing path are outlined below.

### MS/MS data generation

All datasets were generated using Thermo LTQ mass spectrometers. Vendor specific RAW formats were converted to mzXML using the ReadW program (version 4.3.1). One dataset came from PeptideAtlas: *Streptococcus pyogenes* (PAe000284-7). The *Bacillus anthracis* data was published as part of the NIAID PRC and is available at [ftp://141.161.76.88/pub/proteomics_ftp/michigan/uom_09/]. *Yersinia pestis* data have been previously described [17]. The rest of the data were generated at PNNL; these data are available at omics.pnl.gov. PNNL datasets were reported previously [30]. Almost all were generated as part of a broad bottom-up proteomics characterization of their respective organism. Global, soluble and insoluble protein lysates were subjected to 2D LC-MS/MS.

### MS/MS Interpretation

Mass spectra were searched by Inspect [31] against a translation of the genome and subsequently rescored with PepNovo and MSGF [19]. Searches did not include any post-translational modifications, but in accord with Inspect's searching paradigm did not require tryptic specificity. We downloaded genomic DNA from RefSeq, and translated all six frames to generate a protein database. Each stop to stop open reading frame (ORF) was included regardless of coding potential. We concatenated decoy records by shuffling each ORF. We included a decoy database to help measure the relative peptide FDR even though the final scoring algorithm (MSGF) does not use the decoy hits to calculate its probability values. Significant peptide/spectrum matches (PSM) were those with an evalue of e-10 or better, which led to a **peptide level** FDR of ∼0.3%. We note here the distinction between PSM level FDR and peptide level FDR. PSM level FDR is calculated from the collection of all spectra passing filters and the number of those spectra with peptides matching the decoy database. Peptide level FDR is calculated from the list of peptide sequences passing filters (many of which were identified in multiple spectra). This number is always larger than the PSM FDR. For example, in the *Mycobacterium tuberculosis* dataset, 160,795 spectra passed the MSGF filter; 40 spectra identified peptides from the decoy database. Thus the PSM or spectrum FDR is 40/160,795 or 0.024%. Within these spectra, 23,451 peptides are identified, of which 37 come from the decoy database. The Peptide FDR is 0.1%.

Peptide spectrum matches from Inspect and MSGF, as well as the mapped peptide locations for all datasets can be downloaded from the PNNL website: http://omics.pnl.gov/pgp/overview.php.

### ORF Filters

ORF filters are based on the set of peptides within an open reading frame. All confident peptide identifications were mapped onto their genomic location (nucleotide coordinates) and grouped into sets within an ORF. We employ five ORF filters. First we remove low complexity peptides, with >70% glycine and alanine [17]. Next we remove peptides which are more than 750 bp from the next in-frame peptide. We remove ORFs which lack a uniquely mapping peptide or which lack a fully tryptic peptide. Finally, we require two peptides per protein. The interpeptide length filter is designed to overcome weakness in the min-peptide filter. The two peptide rule relies on the low probability of two false-positives falling within the same open reading frame. Yet for high GC genomes there are numerous long open reading frames that are not genic. Thus the likelihood of two peptides within one of these is higher. To determine the average interpeptide distance we sorted all peptides within a current protein annotation and then tallied the nucleotide distance between consecutive pairs. We plotted a histogram of all distances and found that 750 bp was an appropriate cutoff.

### Conflict report

We created a conflict report to describe overlapping (i.e., conflicting) protein annotations. The types of conflict were differentiated by the nucleotide length of the conflict and the biological implication. We distinguished between *annotated protein region* and *proteomic evidence region*. The annotated region was obtained from the RefSeq record. The proteomic evidence region was from the 5′ most peptide through to the stop codon. Conflict levels were defined as:

Level 1 –overlap by less than 10 bp.

Level 2 – overlap by less than 40 bp.

Level 3 – overlap by 40 bp or more. There is proteomic support for only one protein; the unsupported protein contains “hypothetical” in the name.

Level 4 – overlap by 40 bp or more. There is proteomic support for only one protein; the unsupported protein does not contain “hypothetical” in the name.

Level 5 – overlap by 40 bp or more. There is proteomic support for both proteins. The overlap is limited to the annotated region and is not in the proteomic evidence region.x

Level 6 – overlap by 40 bp or more. There is proteomic support for both proteins. The overlap is within the proteomic evidence region.

### Proteogenomic reannotation

Open reading frames passing filters were compared against the RefSeq annotation. ORFs that contained peptides but not a protein annotation were reported as novel proteins. ORFs that have peptides upstream of a protein annotation are reported as ‘new start’ proteins. As a caveat for new start assignments, we required that peptides contain at least two upstream amino acids [17]. We also reported new starts when proteomic evidence was indicative of an alternative start codon TTG or GTG translated as Leucine or Valine. To determine the start site of proteogenomic corrections (novel gene and new start), our overriding choice was to find the upstream start site closest to the peptides found by mass spectrometry, unless blast homology strongly suggested consensus at another start site. We took a conservative approach; it is always easier to add sequence (i.e. extend further upstream) than remove, because of the difficulty of proving negative evidence. For peptides that overlapped pseudogenes, we did not attempt to update RefSeq with a new gene. Additionally, we did not attempt to update a start sites when peptides had no upstream start site.

### In Vivo Cleavage

To report a protein as containing a signal peptide, we started with proteins where the first observed peptide was not tryptic on its N-terminus, and was within 15–50 amino acids of the predicted start site. Between the initial methionine and the first observed peptide is the putative signal peptide. We filtered this set using previously recognized signal peptide characteristics [24]. We required a hydrophobic patch of at least eight contiguous amino acids and examined the signal peptide terminus for the expected cleavage motif. We also required a basic residue between the start and the hydrophobic patch.

Lipoproteins were found in a similar manner, but with a distinct motif L-x-G/A-C, where cysteine forms the first residue of the mature protein and is modified with a lipid. In all instances, however, the peptide identified in MS/MS began immediately after the cysteine. As we did not search for any lipid modifications, we did not expect to find any cysteine-modified peptides. Observed peptides starting immediately after the cysteine were considered evidence for a lipoprotein.

## Supporting Information

Figure S1 **False Positive Peptide at Zero FDR.** The red peptide (GGVGGHLAPDAAAR) is a fully tryptic peptide with an MSGF e value of 7e-17. It lies in a small unannotated open reading frame of 272 amino acids. This ORF has 100% overlap with the current C. crescentus gene CCNA_00970, which is both a well-known gene and also well supported by proteomics. This proposed novel ORF is an example of a false-positive, which is present even at presumed zero FDR.
(TIF)

Figure S2 **Errors in annotation by year.** Novel genes discovered by proteogenomics are plotted by the year that the original annotation was published. The high mark in the dataset (y = 113) is the *Deinococcus* genome, which suffers from significant genome sequence errors (see errata in White et al 1999) likely causing the exceptionally high misannotation rate. Discounting that data point, errors seem to be uncorrelated with year.
(TIF)

Figure S3 **Errors by GC.** Novel genes discovered in proteogenomics are plotted according to the GC content of the genome. There appears to be no strong correlation between high GC and error rate. As with figure S1, the high mark in the data set (y = 113) is believed to be an outlier due to abundant errors in the genome sequence.
(TIF)

Figure S4 **GC content by gene type.** The GC distribution of four gene categories. Grey is the genes for which proteogenomics does not suggest a change. Blue is novel genes. Red is the novel extension to a current gene. Green is the original (now c-terminal) portion of genes that have been extended. In all datasets except the *Cyanobacterium*, the unchanged and novel genes show similar GC content. In *Cyanobacterium*, the novel genes appear to have lower GC. The extensions to current genes (red) show a wider distribution than their parent gene models (green).
(TIF)

Figure S5 **Codon usage.** Codon usage frequencies from all unchanged genes have been dimension reduced to 2D through principal component analysis (see Medigue et al., 1991). The codon frequencies for novel genes were transformed using the same pca vector weighting and mapped in blue on top of the unchanged genes. Codon usage does not appear to be substantively different between the novel and unchanged gene sets. A, *C. crescentus*; B, *C. synechocystis*; C, *D. desulfricans*; D, *L. interrogans*.
(TIF)

Figure S6 **Length comparison.** The length of all genes (grey) has a median of ∼900 nucleotides with a long tail out to 10,000 nucleotides. Novel genes (blue) are on average shorter than the background distribution. However, they are not too short to have fallen below cutoff.
(TIF)

Table S1 **List of organisms presented in this study.** Along with each organism is listed the following information: the percent GC of the genome, the date of genome submission to genbank, the RefSeq accession of the primary chromosome, the genome size in megabases, the number of novel genes discovered through proteogenomics, the number of genes for which proteomics data suggests a new start site, the number of validated signal peptide cleavages, the total number of peptides discovered in the MS/MS data for an organism.
(XLSX)

Table S2 **Gene conflicts.** This table presents the number of conflicting loci, separated by type, for each genome.
(XLS)

Table S3 **Change in conflicted loci according to PSM specificity.** For each organism, the number of conflicts (levels 4,5,6) are shown at two different PSM specificity cutoffs, a loose 5e-08 and the default 1e-10. With the loose cutoff there is often a much larger number of conflicted loci, indicating a high false-discovery at the protein level.
(XLS)
