# Supplementary figures and images for: Proteogenomic Analysis of Bacteria and Archaea: A 46 Organism Case Study
Source: PLoS One. 2011 Nov 17;6(11):e27587. doi: 10.1371/journal.pone.0027587 (PMC3219674; doi:10.1371/journal.pone.0027587)

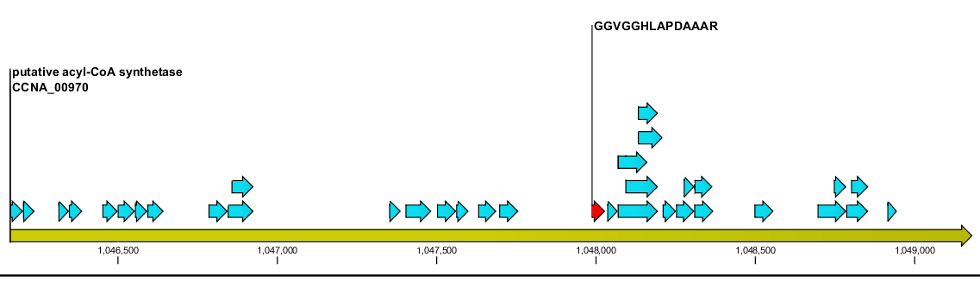

Supplement: Figure S1 — False Positive Peptide at Zero FDR. The red peptide (GGVGGHLAPDAAAR) is a fully tryptic peptide with an MSGF e value of 7e-17. It lies in a small unannotated open reading frame of 272 amino acids. This ORF has 100% overlap with the current C. crescentus gene CCNA_00970, which is both a well-known gene and also well supported by proteomics. This proposed novel ORF is an example of a false-positive, which is present even at presumed zero FDR. (TIF) [file pone.0027587.s001.tif]

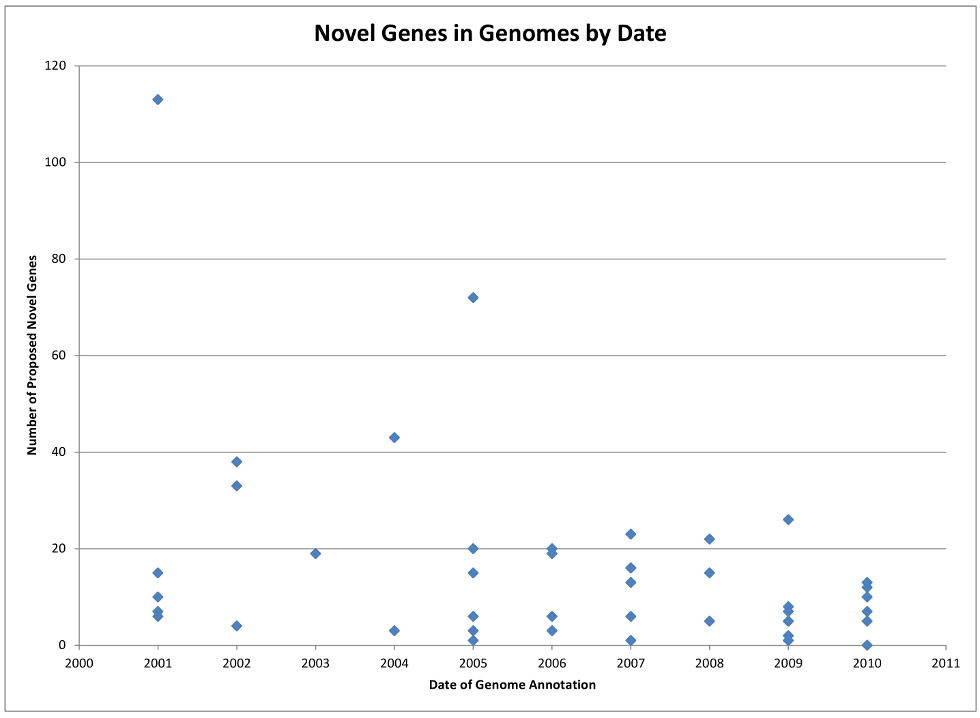

Supplement: Figure S2 — Errors in annotation by year. Novel genes discovered by proteogenomics are plotted by the year that the original annotation was published. The high mark in the dataset (y = 113) is the Deinococcus genome, which suffers from significant genome sequence errors (see errata in White et al 1999) likely causing the exceptionally high misannotation rate. Discounting that data point, errors seem to be uncorrelated with year. (TIF) [file pone.0027587.s002.tif]

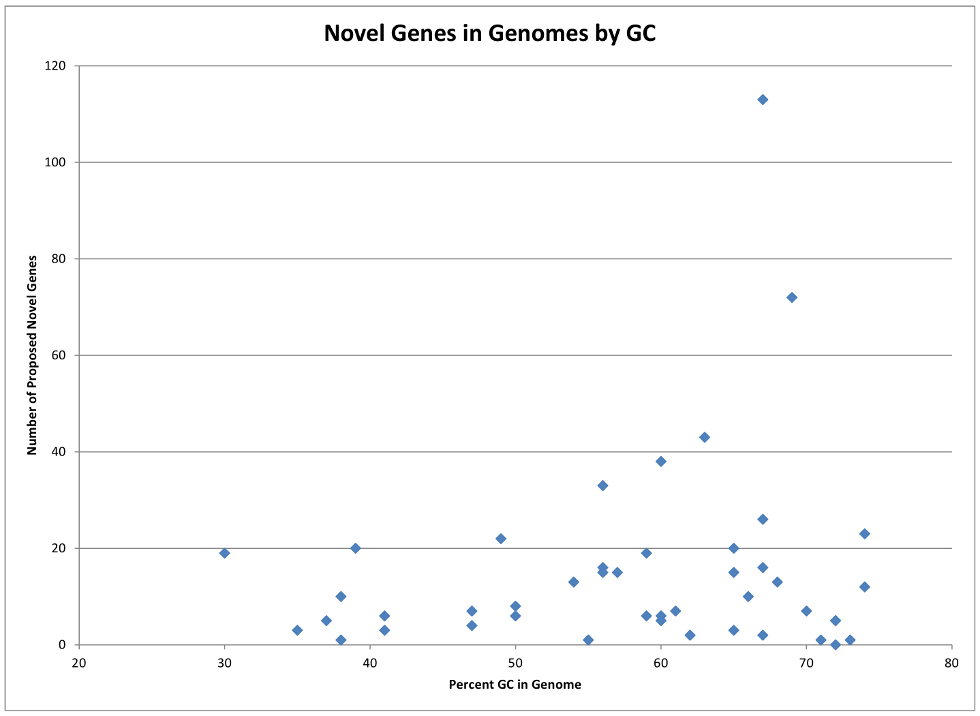

Supplement: Figure S3 — Errors by GC. Novel genes discovered in proteogenomics are plotted according to the GC content of the genome. There appears to be no strong correlation between high GC and error rate. As with figure S1, the high mark in the data set (y = 113) is believed to be an outlier due to abundant errors in the genome sequence. (TIF) [file pone.0027587.s003.tif]

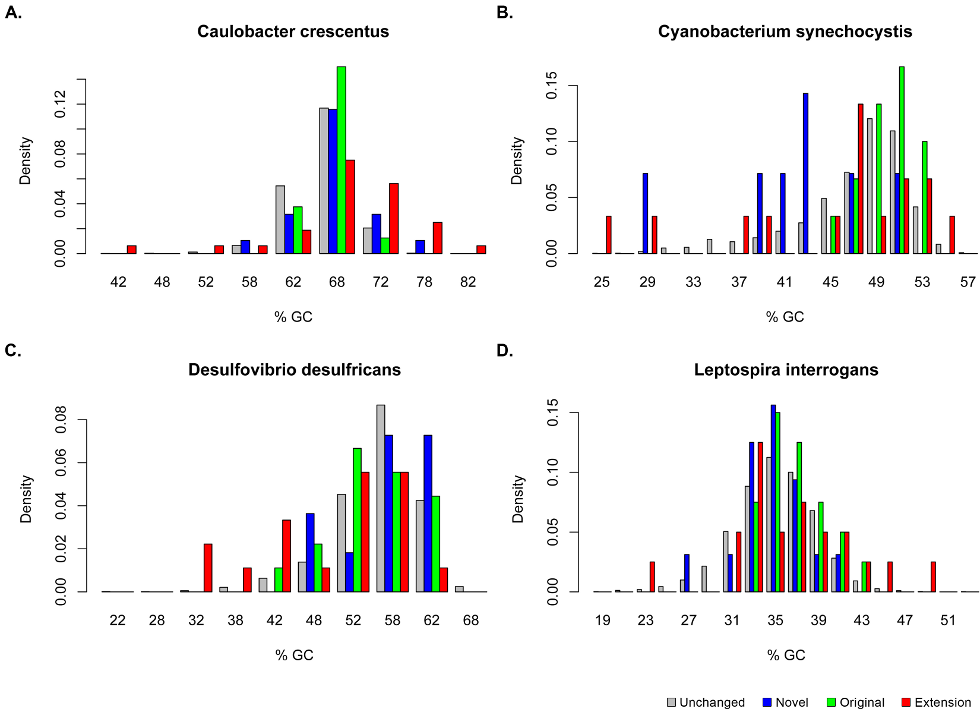

Supplement: Figure S4 — GC content by gene type. The GC distribution of four gene categories. Grey is the genes for which proteogenomics does not suggest a change. Blue is novel genes. Red is the novel extension to a current gene. Green is the original (now c-terminal) portion of genes that have been extended. In all datasets except the Cyanobacterium, the unchanged and novel genes show similar GC content. In Cyanobacterium, the novel genes appear to have lower GC. The extensions to current genes (red) show a wider distribution than their parent gene models (green). (TIF) [file pone.0027587.s004.tif]

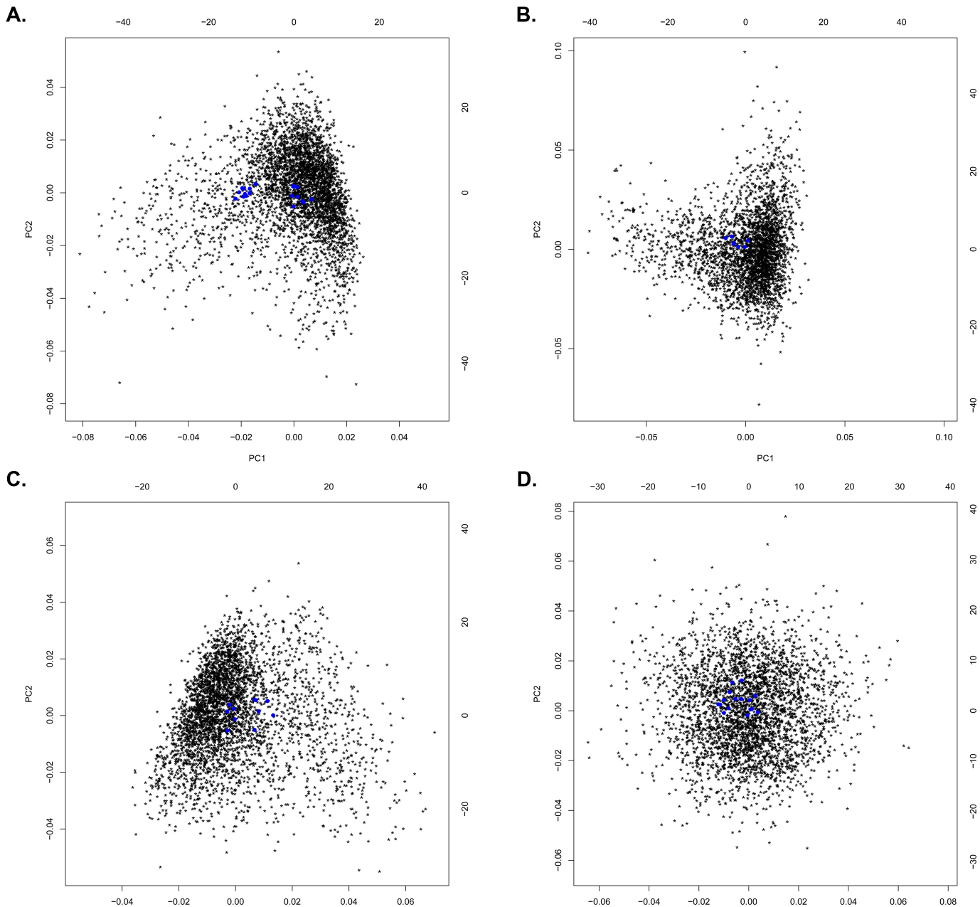

Supplement: Figure S5 — Codon usage. Codon usage frequencies from all unchanged genes have been dimension reduced to 2D through principal component analysis (see Medigue et al., 1991). The codon frequencies for novel genes were transformed using the same pca vector weighting and mapped in blue on top of the unchanged genes. Codon usage does not appear to be substantively different between the novel and unchanged gene sets. A, C. crescentus; B, C. synechocystis; C, D. desulfricans; D, L. interrogans. (TIF) [file pone.0027587.s005.tif]

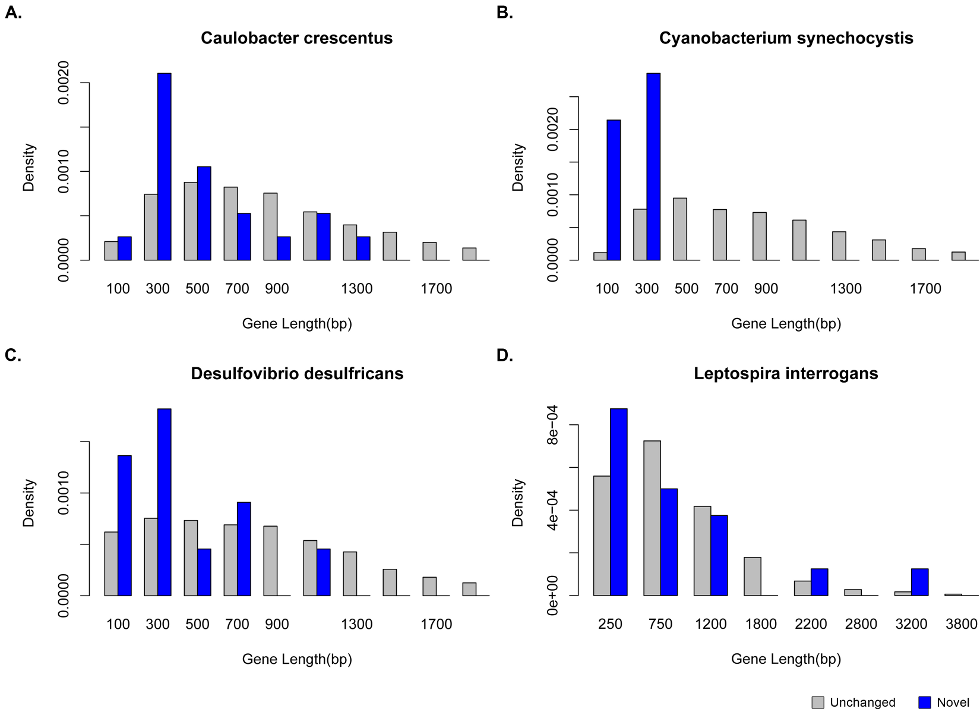

Supplement: Figure S6 — Length comparison. The length of all genes (grey) has a median of ∼900 nucleotides with a long tail out to 10,000 nucleotides. Novel genes (blue) are on average shorter than the background distribution. However, they are not too short to have fallen below cutoff. (TIF) [file pone.0027587.s006.tif]
